# Metabolism and Immune Modulation in Patients with Solid Tumors: Systematic Review of Preclinical and Clinical Evidence

**DOI:** 10.3390/cancers12051153

**Published:** 2020-05-04

**Authors:** Aurora Mirabile, Licia Rivoltini, Elena Daveri, Claudio Vernieri, Roberto Mele, Luca Porcu, Chiara Lazzari, Alessandra Bulotta, Maria Grazia Viganò, Stefano Cascinu, Vanesa Gregorc

**Affiliations:** 1 Department of Medical Oncology, Scientific Institute San Raffaele Hospital, Via Olgettina, 60, 20132 Milan, Italy; chiara.lazzari@hsr.it (C.L.); bulotta.alessandra@hsr.it (A.B.); vigano.mariagrazia@hsr.it (M.G.V.); cascinu.stefano@hsr.it (S.C.); gregorc.vanesa@hsr.it (V.G.); 2 Immunotherapy of Human Tumors, IRCCS National Cancer Institute (INT) and University of Milan, Via Venezian 1, 20133 Milan, Italy; licia.rivoltini@istitutotumori.mi.it (L.R.); elena.daveri@istitutotumori.mi.it (E.D.); 3 Medical Oncology Department, IRCCS IRCCS National Cancer Institute (INT) and University of Milan, Via Venezian 1, 20133 Milan, Italy; claudio.vernieri@istitutotumori.mi.it; 4 IFOM, the FIRC Institute of Molecular Oncology, Via Adamello 16, 20139 Milan, Italy; 5 Nutritionist biologist, Hospital Health Direction, Scientific Institute San Raffaele Hospital, Via Olgettina 60, 20132 Milan, Italy; mele.roberto@hsr.it; 6 Methodological Research Unit, Institute of Pharmacological Research Mario Negri, Via Mario Negri 2, 20156 Milan, Italy; luca.porcu@marionegri.it

**Keywords:** immunotherapy, nutrition, immune response, immune-nutrition, cancer metabolism

## Abstract

Several immunotherapy agents are the standard of care of many solid malignancies. Nevertheless, the majority of patients do not benefit from the currently available immunotherapies. It is therefore of paramount importance to identify the prognostic and predictive factors of tumor response/resistance and to design effective therapeutic strategies to overcome primary resistance and improve the efficacy of immunotherapy. The aim of this review is to underline the influence of the tumor and host metabolism on the antitumor immune response and to discuss possible strategies to improve the efficacy of available treatments by targeting the specific metabolic pathways in tumors or immune cells and by modifying patients’ nutritional statuses. A systematic search of the Medline and EMBASE databases was carried out to identify scientific papers published until February 2020, which reported original research articles on the influence of tumor or host metabolism on antitumor immune response. The literature data showed the key role of glycolysis and mitochondrial oxidative phosphorylation, arginine, tryptophan, glutamine, lipid metabolism and microbiome on immune cell function. Moreover, specific nutritional behaviors, such as a low dietary intake of vitamin C, low glycemic index and alpha-linolenic acid, eicosapentenoic acid, docosahexaenoic acid, ornithine ketoglutarate, tryptophan and probiotic supplementation were associated with the potential clinical benefits from the currently available immunotherapies.

## 1. Introduction

Immune checkpoint inhibitors (ICIs) have revolutionized the treatment of solid and hematologic malignancies and have become a key therapeutic tool for the management of cancer patients. Different monoclonal antibodies targeting the programmed death 1 (PD-1) or PD-1 ligand (PD-L1) are currently used for the treatment of metastatic melanoma, non-small cell lung cancer (NSCLC), classic Hodgkin’s lymphoma, urothelial bladder, renal cell and head and neck squamous cell carcinomas [1,2,3,4,5,6,7,8,9,10]. However, despite the impressive and long-lasting efficacy of these agents in a sizable subset of patients, only approximately 20–40% of patients benefit from ICIs, while in the majority of cases, tumors show primary resistance or undergo tumor progression after the initial response as a consequence of an acquired resistance (secondary resistance) [11]. Enhancing tumor antigenicity, lymphocyte priming and migration, cancer cell killing and the reduction of the immunosuppressive tumor microenvironment (TME) represent promising strategies to boost antitumor immunity and to implement the efficacy of a PD-1/PD-L1 blockade [12,13]. ICI clinical activity might be profoundly shaped by specific metabolic routes. Indeed, systemic metabolism, and in particular specific blood metabolites, such as glucose and amino acids, or components of the gut microbiota, play a crucial role in stimulating or restraining the growth, proliferation and activation of specific immune cell populations in TME [14,15,16,17]. Therefore, modulating systemic (host) or tumor metabolism could impact on local (i.e., through changes in the gut microbiota composition and metabolism) and systemic antitumor immunity, thus potentially affecting the clinical efficacy of ICIs.

Recent studies underlined a key role of the whole organism nourishment and metabolism on immune cell function [14,15]. Good nutritional status is essential for the adequate functioning of the immune system, whereas long-term nutrient deprivation and malnutrition cause functional immune impairment [14,15,16,17]. Several studies demonstrated an association between weight loss or sarcopenia and worse prognosis in cancer patients, in part explained by a dysfunctional immune surveillance [18]. At the same time, an excess of energy intake and specific macro-/micronutrients can directly suppress the function of cytotoxic T lymphocytes, which are essential for mounting an effective antitumor immune response [14,15,16]. For instance, obesity and an excess of calorie intake are associated with increased cancer risk, which likely result from an increased availability of metabolite (glucose, amino acids) and growth factor (insulin, insulin-like growth factor 1 or IGF1) availability to cancer cells or their precursors, coupled with the presence of low-grade chronic inflammation that could impair antitumor immunity [19].

In parallel with the clinical data, the preclinical evidence from immunocompetent mouse cancer models showed synergistic antitumor activity between anti-neoplastic drugs and a broad range of therapeutic strategies aimed at reducing the blood concentration of glucose, amino acids and growth factors [20,21]. Whether these observations rely on the involvement of the immune cells is still to be clarified.

In this scenario, the intestinal bacteria, which are essential in maintaining the physiology of gut functioning and local/systemic metabolism, may also regulate local (in the case of colorectal neoplasms) and systemic antitumor immune responses, this affecting the efficacy of chemotherapy and immunotherapy [22]. Indeed, different intestinal bacterial species can modify the concentration of specific metabolites in the intestinal lumen, thus modulating the type and activation status of several local immune cell populations, including the immune cell populations directly implicated in mounting an effective antitumor response. 

Given the relevance of ICI-based immunotherapy for the treatment of several human cancers, many preclinical and clinical research efforts are presently devoted to enhance their therapeutic efficacy. 

In this perspective, a promising strategy is to investigate the role of modulating specific metabolic pathways in the cells of the immune system and in cancer cells, and in particular to implement both pharmacological and dietary interventions to this aim. This work provides a systematic review on the potential metabolic patterns that affect the immune system functions, and gives an insight into the dietary interventions and possible strategies to improve ICI antitumor efficacy. In particular, we aimed at identifying metabolic pathways that affect the functional status of immune cells in the immune system, as well as metabolic factors that are capable of predicting the antitumor efficacy of currently available immunotherapies. 

## 2. Methods

A systematic search of the Medline and EMBASE databases has been carried out to identify all potentially relevant English language scientific papers reporting original research articles on the interaction between the metabolic pathways and immune cells in cancer patients or preclinical tumor models. Eligible were full text papers written fully in English with available abstracts and at least one of the following characteristics:

- Preclinical studies using a tumor model or clinical studies on oncologic patients that evaluate the influence of nutrition/metabolism on the immune system;
- Clinical and/or preclinical studies about the role of specific metabolites and/or gut microbiota in the immune system homeostasis;
- Studies about how specific metabolites could modify ICI efficacy until February 2020.

Preclinical and clinical studies were excluded if they met at least one of the following criteria:

- No immunomodulation activity endpoint;
- About pediatric or pregnancy patients;
- Reviews;
- Reports;
- Surgical settings;
- Hematological malignancies;
- About carcinogenesis;
- Outcome in healthy people;
- About oncological therapies toxicities;
- About inflammation, infection and cancer prevention.

The following search strings were used in Medline and in EMBASE: “Nutrition OR immune modulation AND tumor”, “nutrition AND immunotherapy” and “microbiota AND immunotherapy”.

Two authors (AM and LP) independently searched articles published in English until January 2020 and selected them according to the inclusion and exclusion criteria. Any disagreements or differences in the selection of the eligible articles were resolved by consultation and discussion with a third assessor (GV). The date last searched was 20 February 2020. A Preferred Reporting Items for Systematic Reviews and Meta-analyses (PRISMA) flow diagram (http://prisma-statement.org/PRISMAStatement/FlowDiagram.aspx. Retrieved 10 February 2020) was created to summarize the systematic review process. Reports of the systematic review have been performed according to the PRISMA (Preferred Reporting Items for Systematic Reviews and Meta-analyses) guidelines [23].

## 3. Results

### 3.1. Selection of Preclinical and Clinical Studies

A systematic search of the Medline and EMBASE databases was carried out to identify eligible research papers. The systematic database search yielded 2144 records, of which 2030 were excluded after reviewing the title and abstract. A total of 114 articles were selected for a full-text review and closer inspection to determine whether they met the eligibility criteria.

**Figure 1:**
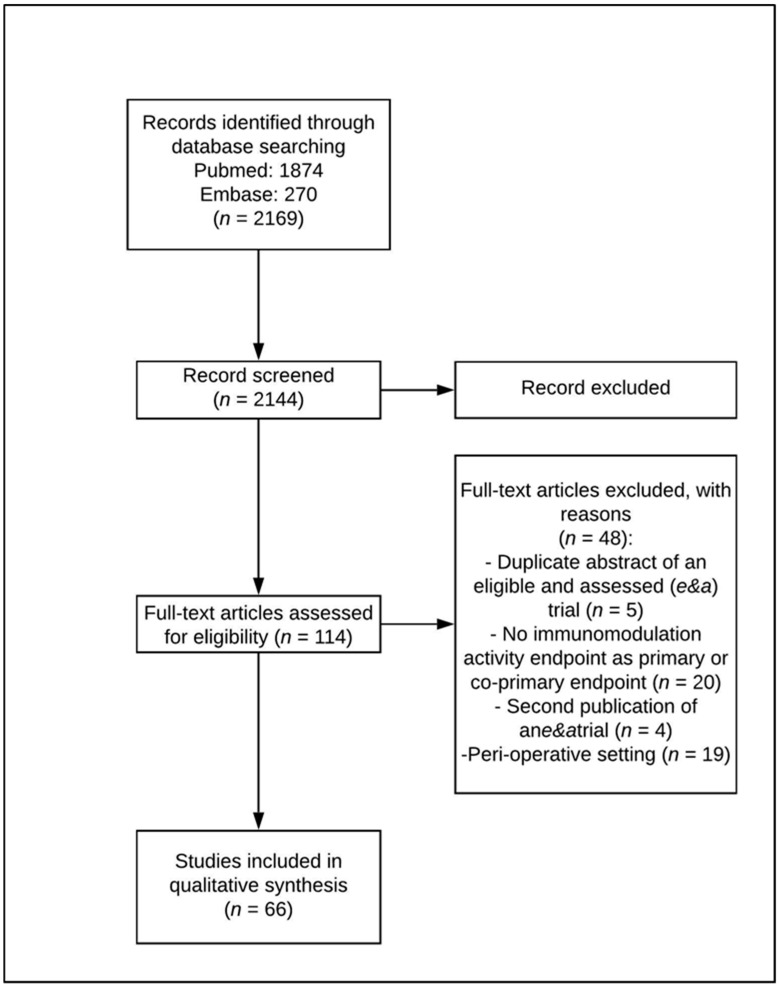
Preferred Reporting Items for Systematic Reviews and Meta-analyses (PRISMA) flow diagram on selection of preclinical and clinical articles.

Forty-eight full-text articles were excluded because they reported the following: (1) any immunomodulation activity endpoint as the primary or co-primary endpoint (*n* = 20, 41.7%); (2) a perioperative setting (*n* = 19; 39.6%); (3) a duplicate abstract of an eligible trial (*n* = 5; 10.4%); and (4) second publications of eligible trials (*n* = 4; 8.3% refer to Figure 1). 

Sixty-six eligible trials in total were finally included in this survey (Table 1), 49 of which were preclinical and 17 clinical.

Unfortunately, 74.2% of these studies reported results of preclinical studies (conducted in in vitro cultured cells or in immunocompetent mice) or consisted of non-randomized clinical studies. Since most of the clinical studies initiated in this research field have not been completed yet, results of our analysis should be considered preliminary, while results of ongoing clinical trials will contribute to clarify the link between cancer patients’ nutrition/metabolism and immunotherapy efficacy. In Table 2, we summarize the available clinical data, which indicate that nutritional behaviors are a potential means to modulate the incidence and progression of cancer, and the response to antitumor treatments. Together, the available evidence indicates that the abundance of energy-rich metabolites (such as glucose, fatty acids or amino acids) and/or trophic factors (such as insulin, insulin-like growth factor and leptin) can affect tumor immuno-surveillance and stimulate the proliferation of cancer cells. 

### 3.2. Glycolysis and Oxidative Metabolism

Immune cells require a large amount of energy units (ATP) and reducing equivalents (NADH, FADH_2_) to guarantee their biological functions; these molecules mainly derive from glycolysis and oxidative phosphorylation (OxPhos) [41,42,43]. Depending on the type of nutrients, oxygen availability and the specific immune cell population, glycolysis and OxPhos can become the predominant way to fuel cell metabolism and to guarantee a proper balance of the intracellular redox status. In conditions of normoxia, the glycolytic pathway converts glucose to acetyl-CoA, which enters the tricarboxylic acid (TCA) cycle to ultimately drive OxPhos and to generate ATP and the reducing equivalents. However, OxPhos also takes part in the final steps of glutamine and fatty acids (FAs) metabolism, which enter the TCA cycle to fuel the energy production and anaerobic pathways. Under hypoxic conditions or during fast replication, immune cells produce ATP prevalently via glycolysis, and convert pyruvate into lactate rather than into acetyl-CoA. Conversely, most cancer cells use glycolysis as the main source of energy and anabolic precursors even in oxygen-rich conditions (Warburg Effect) [41,43,44].

Metabolic and functional activities of different T cell subsets require glucose uptake and metabolism in the glycolysis pathway to sustain cell proliferation and activation upon T cell receptor (TCR) triggering [45,46,47]. Resting naïve T cells mainly rely on oxidative phosphorylation (OxPhos) for their energy demand, while after an antigen encounter, the stimulated T cells rapidly proliferate and undergo metabolic reprogramming by increasing glucose uptake and activating aerobic glycolysis. The glycolytic metabolism is used by different effector T cell subsets, including Th1, Th2 and Th17 CD4 T cells and cytotoxic CD8 T cells, while regulatory T cells (Treg) are less reliant on glycolysis and depend mainly on the mitochondrial oxidative metabolism of lipids [48]. At the molecular level, costimulation of TCR and CD28 induces the activation of the PI3K/Akt/mTOR signaling pathway, which in turn promotes the expression of the *Glut1* gene and the hypoxia-inducible factor 1α (HIF1α) and leads to enhanced glycolysis. Of note, the mammalian target of rapamicin (mTOR) kinase is essential for Th1 and Th17 differentiation, as well as for the inhibition of Treg generation [49]. Furthermore, the transcription factor c-Myc plays an important role in the glycolytic metabolism by up-regulating the expression of the GLUT-1 transporter in activated T cells [50].

Different populations of macrophages preferentially utilize glycolysis or FA oxidation/OxPhos to sustain different cellular functions [51]. In particular, antitumor M1-like macrophages utilize glycolysis to generate ATP, while protumor M2 macrophages preferentially utilize OxPhos [52]. The TCA cycle intermediate succinate plays a crucial role in promoting a macrophage switch from OxPhos to glycolysis, as well as to stimulate the secretion of pro-inflammatory cytokines [51,53]. Succinate promotes the stabilization of HIF1α, which in turn stimulates the expression of the proinflammatory cytokine IL-1β [51]; in other cellular contexts, HIF1α can promote the expression of myeloid cells’ immunosuppressive molecules, such as miR-210 and PD-L1 [52,54]. Therefore, depending on the context, the immunomodulatory role of HIF1α can change, with more prominent pro-inflammatory or anti-inflammatory functions in different contexts [52,55,56,57,58,59,60]. Succinate can also have pro-inflammatory effects through ligating the succinate receptor 1 to increase the dendritic cells’ (DC) chemotaxis to enhance DC-induced T cell responses [59,60]. 

Cancer cells reprogram their metabolism by upregulating glucose uptake, which results in a glucose- deficient TME [45,46,47]. Glucose deficiency in a TME can directly inhibit glycolysis in immune cells, thus impairing antitumor immune responses [61,62]. Cytotoxic functions of tumor-infiltrating effector lymphocytes are particularly affected by a lowered glucose concentration in a TME [45,63,64]. On this matter, low glucose levels activate oxidative metabolism in macrophages and promote the M2-like phenotype, featured by anti-inflammatory and immunosuppressive functions [41,65,66]. Glucose deprivation in a TME enhances the IC-mediated negative signals, which in turn suppress the TCR and increase the intra-tumor accumulation of immunosuppressive Treg cells. 

Similar to the case of resting T cells, the balance between glycolysis and OxPhos affects the dendritic cells’ (DC) function.

Indeed, resting DCs depend on OxPhos for energy generation, but are able to rapidly switch to a glycolytic program after activation [67]. 

Based on this evidence, promoting glucose utilization by tumor-infiltrating lymphocytes and M1 macrophages and DCs could boost the activity of anticancer immunity without over-feeding the tumor. However, the most effective way to improve glucose utilization by immune cells is far from being established. Indeed, any dietary intervention aimed at inhibiting tumor glycolysis by reducing the availability of glucose to cancer cells, including cyclic fasting or fasting-mimicking diets (FMDs), would also increase the competition for residual glucose molecules between cancer and immune cells in a TME, thus potentially reducing the glucose provision to the T cells, M1 macrophages and DCs [38,46,68,69,70,71]. On the other hand, increasing the blood concentration of glucose with the aim of stimulating cytotoxic T lymphocytes could overfeed the tumor, thus boosting its ability to remove glucose in a TME. 

However, these considerations are limited by the lack of knowledge of the effect of lowering or increasing the blood glucose concentration in a TME, and in particular, in the extracellular environment of cancer cells and of specific intratumor immune cell populations.

Pharmacological strategies that more selectively activate or inhibit glycolysis in cancer cells or in specific immune cell populations could be more effective to boost antitumor immunity. 

Human studies suggested that the chronic administration of some types of anti-hyperglycemic medications (e.g., metformin) or natural polyphenols (such as resveratrol) can reduce cancer risk [72,73,74], but few studies have investigated their interaction with immunotherapy. One retrospective cohort study reported an improved PFS and OS, but they were not yet statistically significant in patients with metastatic malignant melanoma who receive the metformin in combination with ICI compared with ICI alone [75]. Another trial is currently investigating metformin in combination with immunotherapy in NSCLC [76]. Concerning the resveratrol impact on ICI efficacy, no clinical trials are available, but trials in cancer-free [77,78,79] and tumor-bearing [79,80,81] subjects suggest that it can improve T cell function and favor an anti-cancer response. 

One promising strategy to specifically modulate the rate of glucose utilization in lymphocytes and tumor cells consists in selectively inhibiting glucose uptake in cancer cells; to this aim, selective GLUT1 inhibitors have been tested in preclinical experiments; however, they were associated with excessive toxicity to be used in the clinic [82]. 

Glucose-derived phosphoenolpyruvate (PEP) affects calcium signaling and promotes the antitumor activity of T cells, while low intracellular PEP levels result in an increased Ca^2+^ uptake into the endoplasmic reticulum, inhibition of the nuclear factor of activated T cell (NFAT) and reduced T cell effector function [45]. These findings support a direct link between the activation of glycolysis and the generation of functional T cell responses. 

In conditions of limited glucose availability, increasing intracellular PEP concentrations could facilitate Ca^2+^ signaling and promote pro-inflammatory and antitumor functions in lymphocytes. PEP levels are balanced by enolase-mediated PEP formations and the pyruvate kinase-mediated (PKM) conversions of PEP to pyruvate. Therefore, a pharmacological inhibition of PKM1/2 could increase PEP levels and restore normal Ca^2+^ signaling, thus stimulating antitumor immunity [83]. Studies conducted in tumor-bearing immunocompetent mice have recently demonstrated that a glucose-depleted TME limits aerobic glycolysis in tumor-infiltrating T cells, which suppresses anticancer effector functions; however, inducing the expression of the PEP carboxykinase 1 (PCK1) in tumor- infiltrating CD4+ T cells is sufficient to restore PEP levels and promotes T cell signaling through the TCR [45]. 

In addition, the indirect effects of anticancer therapies on the metabolic status of intra tumor immune cells could also play an important role. For instance, the combination of chemotherapy or radiotherapy with immunotherapy improves the anticancer efficacy of immunotherapy by inducing immunogenic cell death and reducing the glucose demand by cancer cells; in these conditions, glucose is preferentially utilized by tumor-infiltrating antitumor lymphocytes, thus boosting their antitumor activity [54,84]. In a recent research, the glycolytic enzyme enolase-1 was implicated in controlling FOXP3 splicing in human Tregs and in inhibiting their immunosuppressive functions [85]. 

Together, the available preclinical evidence suggests that reducing the glucose availability in a TME can reduce the glucose availability to glycolytic cancer cells and immune suppressive Tregs but, at the same time, it can impair the proliferation and activation status of antitumor T lymphocytes and pro-inflammatory M1 macrophages and DCs. Therefore, the final outcome (pro-tumorigenic vs. anti-tumorigenic) of the glucose modulation in a TME could depend on different factors, including the tumor type, baseline functional state of antitumor immunity and the type of systemic treatment that is used. From this perspective, ongoing clinical studies are testing standard immunotherapy agents in combination with metformin (in advanced melanoma and NSCLC) or cyclic FMD to reduce glucose utilization in cancer cells (through metformin or FMD), while at the same time activating cytotoxic lymphocytes through the inhibition of PD-1 [55,86,87]. 

Although the activity of metformin on tumor metabolism at clinical doses is still controversial, it could produce different anticancer effects. By affecting the host (systemic) metabolism, metformin could impair tumor cell growth and proliferation through reducing the blood glucose, insulin and IGF-1 levels, as well as through affecting the blood concentration of NFkB and pro-inflammatory cytokines and improving the anticancer immune response. Metformin can also act through direct mechanisms, i.e., by inhibiting the mTORCq pathway, but also affecting the intracellular folate levels, c-MYC activation, gluconeogenesis, liver glucose secretion and also NFkB, enhancing the p53 phosphorylation and AMPK-independent effects with the increase of mTORC1, autophagy and apoptosis of cancer cells and the reduction of ROS and cyclin D1 [88,89].

**Figure 2:**
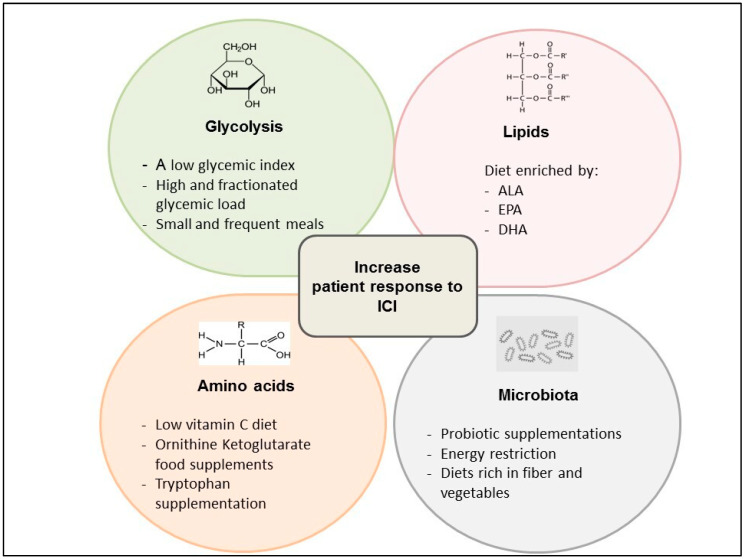
Clinical ongoing approaches based on metabolic modulation as a strategy to improve the response to immune checkpoint inhibitors (ICI).

Recent evidence show that a low glycemic index, coupled with an overall high and fractionated glycemic load (small, frequent meals) diet, could balance the effect of the activation of the glycolytic pathway, leading both to immune system activation through the modulation of the T cell response and the stimulation of tumor growth [90]. In fact, T cells can take advantage of a lower proliferation rate compared with the most aggressive tumors, in association with ICIs, limiting the insulin anabolizing effects (Figure 2).

### 3.3. Amino Acid Metabolism

Arginine, tryptophan and glutamine are crucially implicated in mounting an effective antitumor immune response and their extracellular and intracellular concentration could affect the efficacy of currently available immunotherapies [25,26,27,28]. Two enzymes take part in arginine catabolism, namely arginase-1 and an inducible nitric oxide synthase (iNOS). Arginase-1 catalyzes the conversion of arginine ornithine [91,92,93,94,95], and is frequently upregulated in tumor-associated macrophages (TAMs) and myeloid-derived suppressor cells (MDSCs); moreover, it promotes cancer cell proliferation through the production of polyamines [96], as well as through impairing the T cell receptor (TCR) function [96,97] and T cell differentiation [98]. On the other hand, iNOS catalyzes the conversion of arginine to citrulline and nitric oxide (NO), which is required for the different steps of T cell activation. Therefore, different branches of arginine catabolism can have a different impact on antitumor immunity activation. Owing to the immune-suppressive and pro-tumorigenic role of arginine metabolism through the arginase pathway (but not the iNOS pathway), pharmacological inhibitors of arginase are being tested, alone or in combination with chemotherapy or ICIs, to boost antitumor immune responses [99,100]. Therefore, arginase depletion may also affect the macrophage function within the TME. Arginase inhibitors could establish an antitumor immune response by preventing an ornithine and urea formation, while concomitantly promoting an NO formation and M1 macrophages activation [65,66,101,102,103].

In 2016, an important study published by Geiger at al. found several changes in the metabolic pathways such as the role of L-arginine in controlling glycolysis and mitochondrial activity, in enhancing T cell survival by the interaction with transcriptional regulators and in promoting central memory-like T cells generation with a powered anti-tumor activity [104].

Tryptophan is an essential amino acid for the survival, proliferation and activation of lymphocytes. Several cell populations in a TME, including tumor cells and MDSCs, express indole amine-pyrrole 2,3-dioxygenase (IDO), which catalyzes the first biochemical step in the cascade, leading to the conversion of L-tryptophan to kynurenine. IDO-induced catabolism of tryptophan impairs the glycolysis and mTOR complex 1 (mTORC1) function in activated T cells [105,106,107], thus resulting in the inhibition of effector T cell responses and contributing to enhance immunosuppressive Tregs [108,109,110]. In addition, an increased intratumor kynurenine concentration displays immune suppressive activities on T cells and NK cells [63,105,106,107,111,112] and acts as an endogenous ligand of the Aryl hydrocarbon receptor, which can stimulate Treg cells [104]. 

Based on this evidence, the pharmacological inhibition of IDO1 in a TME could contribute to relieve immune suppression by enhancing the activity of NK and T lymphocytes, while inhibiting Tregs. 

The IDO1 inhibitor epacadostat has demonstrated good tolerability [24,113,114] and is under investigation in combination with different anticancer treatments [115,116]. In particular, IDO inhibitors have been tested in a phase I/II clinical trial in combination with ipilimumab (anti-CTLA4) in patients with metastatic melanoma [117]. Quite disappointingly, in the recently published ECHO 301 trial, the epacadostat–pembrolizumab (anti PD-1) combination failed to demonstrate improved patient PFS when compared with pembrolizumab monotherapy [118,119]. 

Owing to the importance of tryptophan in stimulating antitumor immunity, dietary supplementation of tryptophan could result in an increased tryptophan concentration in a TME and in a reduced conversion to kynureine. Tryptophan is found in high concentrations in dried spirulina and goa beans, which can be added to a standard healthy diet as an alternative to protein enriched food [112]. 

However, no clinical trials to test the efficacy of dietary tryptophan supplementation in boosting antitumor immunity functions and/or improving the clinical efficacy of immunotherapy have been published yet [24].

Glutamine is a non-essential amino acid, whose uptake and metabolism are frequently upregulated in cancer cells. Indeed, most cancer cells use glutamine as a source of energy and anabolic precursors. In particular, glutamine-dependent malignancies typically upregulate the glutaminase 1 (GLS1) enzyme, which converts glutamine to glutamate and α-ketogluratate (α-KG); then, α-KG enters the KREBS cycle, where it is used as an anabolic precursor for the production of a citrate (the precursor of FAs and cholesterol) and other KREBS cycle intermediates [120]. 

However, glutamine also sustains lymphocyte proliferation and stimulates the production of cytokines by the activated lymphocytes and macrophages [121].

In particular, an impaired glutamine metabolism inhibits the effector T cell activation and maintains Treg differentiation by decreasing the mTORC1 activity and c-Myc gene expression [122]. Recent findings showed that GLS has a distinct role to promote Th17 but constrain Th1 and the cytotoxic effector T cell differentiation through the IL-2-mediated mTORC1 signaling pathway activation [123].

Macrophage-mediated phagocytosis also depends on the glutamine availability, and recent evidence also linked glutamine metabolism to TAMs functions [121].

In synthesis, glutamine is essential for the activation of cells involved in both innate and adaptive immunity. Based on this evidence, GLS1 inhibitors (e.g., CB-839) that are being investigated to inhibit glutamine utilization in cancer cells could potentially hamper the activation of antitumor immunity through the inhibition of glutamine metabolism in both T cells and macrophages [124,125,126,127]. However, data from a phase I/II trial indicate that CB-839 is capable of reversing the acquired resistance to PD1/PD-L1 inhibitors in advanced melanoma, NSCLC and renal cell carcinoma, with significant rates of disease stabilizations or tumor regressions [128]. While these findings need to be confirmed in larger ongoing and future studies, it is reasonable to hypothesize the differential effects of GLS-1 inhibitors in cancer cells and immune cells. Indeed, since many cancer cells express GLS-1 at much higher levels than normal cells do [129], GLS-1 inhibition in cancer cells could increase the glutamine concentration in a tumor microenvironment and human plasma, with the result of stimulating antitumor T lymphocytes and promoting anticancer immunity.

Reducing the dietary intake of glutamine could also result in impaired glutamine utilization in cancer cells; however, since glutamine is present in almost each protein-containing food, reducing its dietary intake is a difficult goal. For this reason, pharmacological inhibitors of glutamine utilization could be a more feasible and potentially more effective strategy to inhibit glutamine metabolism in highly glutamine-dependent cancer cells, while also promoting its utilization in antitumor immune cells. In addition, ornithine ketoglutarate food supplements, which increase the glutamine concentration in blood, could produce synergistic antitumor effects in combination with the GLS1 pathway inhibitors, which mainly inhibit the glutamine catabolism in GLS1-overexpressing cancer cells but not in immune cells (Figure 2) [130]. 

It has been recently observed that, in the absence of extracellular lipids, ascorbic acid inhibits nitrosative stress by stimulating the conversion of nitrosating species to NO [131]. In these experimental conditions, ascorbic acid reduced the amount of N-nitrosodimethylamine formation by 5-fold, N-nitrosomorpholine by more than 1000-fold and totally prevented the formation of N-nitrosodiethylamine and N-nitrosopiperidine. In contrast, in the presence of a 10% extracellular concentration of lipids, the ascorbic acid increased instead of reduced the amount of N-nitrosodimethylamine, N-nitrosodiethylamine and N-nitrosopiperidine by approximately 8-, 60- and 140-fold, respectively. Since lipids are physiologically present in human blood (as free-fatty acids or as embedded in plasma lipoproteins), these data indicate that reducing the blood concentration of ascorbic acid by reducing vitamin C dietary intake could prevent the potentially detrimental effects associated with Arginase-1 inhibitors, which increase the production of carcinogenic NO derivates [98,105,131]. 

### 3.4. Lipids Metabolism

The balance between fatty acids (FA) synthesis and FA oxidation controls the differentiation of different T cell subsets. FA synthesis supplies lipid-derived membrane structures during cell proliferation and is necessary for activated effector T cells, while the catabolic FA oxidation mainly provides ATP to sustain the energetic needs of Treg and memory T cells [132,133]. 

Overall, FA oxidation promotes immunosuppressive functions in different tumor-infiltrating cells, such as Treg, MDSCs and TAMs. For instance, immune suppressive Tregs express low concentrations of the glucose transporter Glut1, and mostly rely on FA oxidation for their bioenergetic needs [134]. The activation of an AMP-activated protein kinase (AMPK) and the consequent inhibition of mTOR may play a crucial role in promoting FA oxidation [42]. AMPK is typically activated in conditions of low intracellular ATP concentration; once activated, it orchestrates metabolic responses leading to the inhibition of energy-consuming anabolic processes, such asan mTORC1-induced protein and FA synthesis, and to a concomitant activation of FA oxidation and autophagy. Since AMPK activation and mTORC1 inhibition contribute to stimulate lipid oxidation in Tregs, pharmacological therapies that activate AMPK, such as the antidiabetic metformin, or pharmacological inhibitors of mTORC1, such as the antitumor compound everolimus, could result in Tregs stimulation, thus restraining T cell-mediated immune responses [134]. 

The FA arachidonic acid (AA) is the precursor of prostaglandins, which play a crucial role in modulating systemic immunity, including antitumor immunity. In particular, prostaglandin E2 (PGE2) has been implicated in reprogramming antitumor M1 macrophages to pro-tumor M2 macrophages [125]. In more detail, PGE2 enhances STAT3 activation, induces M1 to M2 macrophage polarization [135], suppresses cytokine production by natural killer (NK) cells and induces Foxp3 expression in naïve T cells, which acquire Treg-associated immunosuppressive functions [136]. Therefore, PGE2 and the sphingolipid molecule sphingosine-1-phosphate (S1P), which are produced by cancer cells or TAMs, mediate the immunosuppressive and metastasis-promoting functions [126,136,137,138]. Consistently, the blockade of the PGE2-producing enzyme, microsomal PGE2 synthase 1 (mPGES1), and cyclooxygenase-2 (COX-2) promoted a reversion of M2-M1 polarization of TAMs in an *Apc*^min/+^ colon cancer model [136], while COX-2 inhibition resulted in reduced PD-L1 expression in bladder cancer [90].

The adipocytes-related hormone leptin physiologically regulates systemic metabolism and influences the activity of the immune system [111]. In particular, leptin modulates the phagocytic functions of macrophages and pro-inflammatory cytokine production, as well as the number and function of T cells. Leptin deficiency has been associated with the loss of innate and adaptive immunity [139]. For these reasons, changes in systemic metabolism, resulting in prolonged leptin reduction, could negatively impact on the function of antitumor immunity.

In synthesis, different lipid metabolic pathways or specific lipid mediators can promote or suppress the activity of specific immune cell populations. Therefore, they are potential targets for pharmacological inhibition. Fatty acid beta-oxidation inhibitors, such as inhibitors of carnitine palmitoyltransferase 1 (CPT1), or inhibitors of PGE2 biosynthesis, such as COX2 inhibitors, could be potentially used to boost the immune system by targeting lipid metabolism.

Diets poor in the total amount of fats can boost the efficacy of several anticancer treatments through reducing the body weight and visceral fat protumor effects. Polyunsaturated fatty acids (PUFA), including eicosapentenoic acid (EPA), alpha-linolenic acid (ALA) and docosahexenoic acid (DHA), have been associated with health benefits, at least in part as a result of their anti-inflammatory effects [25,26,27,28,40]. It is well established that dietary fat composition plays an important role in biological processes: for instance, the omega-3 (n-3) PUFAs, EPA and docosahexaenoic acid (DHA) compete with the n-6 PUFA for enzymes promoting the n-6 PUFA conversion into pro-inflammatory and immune-suppressive prostaglandins, which reprogram M1 macrophages to protumor M2 macrophages, as previously discussed. For these reasons, increasing the absolute or relative (n-3/n-6 ratio) dietary intake of EPA and DHA, alone or in combination with PGE2 inhibitors, could result in enhanced antitumor immunity [139,140] (Figure 2).

### 3.5. Microbiota

The highly metabolically active gut microbiota has recently emerged as a crucial player in modulating the adaptive and innate immune functions at the local and systemic levels [22]. The available evidence supports the conclusion that specific dietary regimens/interventions, alone or combined with microbial supplements (probiotics) validated and approved by regulatory authorities, could boost the antitumor activity of currently available immunotherapy strategies. 

However, only a relatively small number of randomized, clinically controlled trials employing dietary interventions aimed at modifying the gut microbiota composition or metabolism have been published so far [29,30,31,32,33,34,35,36,37]. Collectively, these studies have shown that energy restriction and diets rich in fibers and vegetables are associated with gut microbial changes that could enhance the efficacy of standard anticancer treatments, including immunotherapy [22]. Regarding the impact of specific oral probiotics, the administration of *Bifidobacterium* supplements modulated the activation of DCs and improved the function of tumor specific CD8+ T cells [141] (Figure 2). In tumor-bearing mice, *Bifidobacterium* supplementation improved tumor control similarly to anti–PD-L1 immunotherapy, while combining *Bifidobacterium* supplementation and anti–PD-L1 therapy resulted in synergistic antitumor activity and an almost complete inhibition of in vivo tumor growth [142]. Similarly, studies conducted in both humans and mice showed that specific bacterial species of the gut microbiota potentiate the antitumor effect of monoclonal antibodies that inhibit CTLA-4. T cell responses specific for *B. Thetaiotaomicron* or *B. fragilis* were associated with the efficacy of the CTLA-4 blockade, and the introduction of *B. fragilis* into germ-free mice sensitized to murine neoplasms to CTLA-4 treatment, which was ineffective in germ-free animals [142,143,144]. 

Recent preclinical studies have linked the composition of gut microbiota, or their modifications occurring after the administration of broad-spectrum antibiotics, to the anticancer activity of specific cytotoxic or immunotherapy agents [29,145]. Moreover, the use of antibiotics during immunotherapy in cancer patients has been associated with a lower PFS and OS [29,36,146]. Therefore, modulating the gut microbiota composition, and in particular the equilibrium between the different bacterial species, can affect the antitumor activity of several anticancer treatments, including immunotherapy. These data reflect the modulatory effects of specific bacterial species on local and systemic immunity.

Consistent with the impact of gut microbiota composition on antitumor therapy efficacy, a recent report evaluating gut microbiota in metastatic melanoma patients treated with PD-1 ICIs showed that the composition of the gut microbiota was significantly different in patients responding or not responding to the treatment. In responding patients, the tumor immune infiltrate and the abundance of specific bacteria populations were significantly higher [90,140]. Gut microbiota composition has been also found to modulate intestinal adverse events during ICI treatment [36].

Since broad-spectrum antibiotics can rapidly deplete up to 30% of total intestinal microbes, the impact of antibiotic intake on immunotherapy efficacy was retrospectively evaluated in patients with advanced renal cell carcinoma, urothelial carcinoma and NSCLC. Notably, PFS and OS were significantly shorter in users (within 2 months from immunotherapy initiation) when compared with non-users (3.4 vs. 5.2 months for PFS, respectively; and 12.2 vs. 20.8 months for OS) [147].

Of note, gut microbiota abundance, composition and diversity is dynamically modulated by the type and amount of total calorie intake and macro-nutrient composition of daily life. For instance, diets based on animal products increase the abundance of bile-tolerant microorganisms, while reducing bacteria that metabolize dietary plant polysaccharides [46]. Most of the available evidence in this field derives from preclinical studies, and only recently have human studies been initiated to investigate the correlation between the composition of gut microbiota and metabolic functions in the perspective of defining a tumor-preventive or curative strategy [67,148,149,150,151,152,153,154]. For instance, oral probiotic supplementation has been tested in colorectal cancer (CRC) patients and resulted in the reduction of CRC-associated bacteria in the fecal microbiota upon probiotic intervention [63,155].

Current research also focuses on strategies to enhance the efficacy of immunotherapy based on the gut microbiota composition. In this respect, heterologous fecal microbiota transplantation has been recently trialed for the manipulation of microbiomes from responder to non-responder melanoma patients undergoing immunotherapy [156].

## 4. Discussion

In the last two decades, preclinical and clinical studies convincingly demonstrated that different metabolic pathways are implicated in modulating specific immune cell subsets involved in immune surveillance and antitumor immune response [14,157]. In this systematic review, we discussed the mechanisms through which changes in systemic metabolism can affect antitumor immune response. Moreover, we reviewed the available preclinical and clinical evidence linking specific metabolic pathways to antitumor immunity activation and immunotherapy efficacy, and are expected to yield precious information to clarify the impact of targeting the host or tumor metabolism on the activation of antitumor immunity and on the efficacy of currently available immunotherapy options (Table 3).

At the same time, there is evidence that immunotherapy agents that have demonstrated to be effective in specific cancer patient populations also modulate immune cell metabolism, and that this modulation could contribute to their antitumor activity. For instance, anti-PD-1 and anti-CTLA-4 monoclonal antibodies can reactivate glycolysis and other metabolic pathways in exhausted T cells, thus promoting their activation and antitumor functions [22,158,159].

The binding of PD-1 on T lymphocytes and with PD-L1 on tumor or myeloid cells stimulates glucose uptake and glycolysis by cancer cells and reduces its availability to lymphocytes within a TME [46,160]. By blocking these interactions, anti–PD-1, anti–CTLA-4 and anti–PD-L1 immunotherapies promote glycolytic metabolism in tumor-infiltrating T cells and improve their antitumor functions. 

The anticancer activity of glycolytic compounds is in part mediated by their interference with systemic or intra-tumor metabolism. Since interfering with specific metabolic pathways in a TME, including arginine, tryptophan and glutamine metabolism, could be associated with immunomodulatory and antitumor effects, the combination of anti-PD1/PD-L1 therapies with arginase, IDO1 or GLS1 inhibitors could improve their efficacy or revert secondary resistance [161,162]. Finally, dietary lifestyle appears to be a major regulator of the gut microbiota. 

The microbiome is responsible for the tolerance establishment of commensal bacteria and oral food antigens and its metabolites, as short-chain FAs (acetate, propionate and butyrate) have a role in modulating local immunity.

The gut microbiome regulates local adaptive immunity by inducing proinflammatory T_H_17 and T_reg_ and influences innate immunity by regulating neutrophil aging, which is implicated in the pathogenesis of several inflammatory diseases [145]. Neutrophil aging occurs via Toll-like receptors and MyD88 signaling pathways, and results in impaired neutrophil migration and pro-inflammatory properties. The depletion of the microbiota significantly reduces the number of circulating neutrophils and improves the pathogenesis of organ damage in models of sickle-cell disease or endotoxin-induced septic shock. 

While active immunity is essential to combat bacterial infections, uncontrolled immune responses can have dire consequences, including life-threatening autoimmune diseases. Indeed, one of the physiological functions of immune tolerance consists in its ability to maintain a commensal microbiota consisting of a multitude of foreign microorganisms. 

Nutritional patterns that affect microbiota composition and metabolism, such as the Mediterranean diet and low ketogenic diets, have been shown to affect immune system function and to reduce the risk of developing several cancers, as well as to reduce the mortality associated with them and promote eubiosis instead of dysbiosis, which is associated with Western and hyper caloric diets. Some particular probiotic supplementations (*Bacteroides, Clostridium, Faecalibacterium, Eubacterium, Peptidococcus, Peptidostreptococcus* and *Bifidobacterium*) associate with the following: (1) the restoration of innate and adaptive immunity; (2) the correction of the altered intestinal microbiota; (3) T cell differentiation toward Tregs and Th2 phenotypes; (4) the anti-inflammatory activity; and lastly (5) the stimulation of antimicrobial proteins [140,163].

In summary, preclinical studies indicate that metabolism influences the tumor response to immunotherapy. Clinical prospective studies are needed to confirm this hypothesis and determine which metabolic elements are predictive of patients’ outcomes according to the tumor site and the immunotherapy administered. Additionally, the impact of nutrients like vitamins and micronutrients, such as zinc or magnesium, on the stimulation of the immune system, such as patients’ status in terms of cachexia, sarcopenia, lean body composition, body mass index, skeletal muscle mass index and mini nutritional assessment (MNA) score, should be investigated. Finally, while several studies are testing the anticancer activity of dietary and pharmacological metabolic interventions, the effect of these experimental therapies on the number and activation status of specific immune cell populations should be carefully evaluated, in order to define their role on the antitumor immune system activation and restraining immunosuppressive populations.

## 5. Conclusions

In this systematic review, we highlighted the potential therapeutic impact of targeting specific metabolic pathways and/or of modifying the quantity and quality of nutrient intake and/or of the macro- and micronutrient content in daily life, to stimulate anticancer immunosurveillance and to enhance the antitumor efficacy of the currently available immunotherapies in cancer patients. 

## Figures and Tables

**Table 1:** Studies Characteristics.

Mechanisms	Studies *n* = 66	
	49 Preclinical	17 Clinical
**Glycolysis and Oxidative**	15 (22.7%)	2(3%)
**Arginine, Tryptophan and Glutamine**	18 (27.3%)	1 (1.5%)
**Lipids**	3 (4.5%)	1 (1.5%)
**Microbiota**	3 (4.5%)	11 (16.6%)
**Mixed**	10 (15%)	4 (6%)

**Table 2:** Summary of publications about cancer patients supplemented with macro and micronutrient and their effect on immune system.

Trial	Type of Tumor	Number of Patients	Topics	Evidences	Analyzed Parametres	Results
Beatty et al [24]	Colorectal/Melanoma	52	IDO1 inhibitor	Phase 1	Toxicity Objective responses	Well tolerated. No objective responses. SD lasting ≥ 16 weeks in 7/52 patients.
Machon et al. [25]	Head and neck	31	Aminoacids, vitamins, fatty acids, ribonucleic acids, antioxidants	Observational	Inflammatory/oxidative stress	Decreased hs-CRP (9.8 vs. 3.2, *p* = 0.002) and α-1 acid glycoprotein (1.2 vs. 1.0, *p* = 0.020)
Sunpaweravong et al. [26]	Esophageal	71	Arginine, EPA, DHA and nucleotides	Randomized	Immune cells	Decreased CRP (*p* = 0.001) and TNF (*p* = 0.014)
Maruyama et al. [27]	Gastric and esophageal cancer	22	Arginine, fatty acids and nucleotides	Randomized	Immune cells	Increased Th17 (9.0 ± 2.2 vs. 14.4 ± 3.5%)
Talvas et al. [28]	Head and neck and esophageal	28	Arginine, fatty acids and glutamine	Double blind	Immune cells	Maintained LT4/LT8 counts ratio (2.47 ± 0.31 vs. 1.95 ± 0.20); Decreased PGE2 (66 ± 16 vs. 107 ± 16, *p* < 0.05); Increased IFNγ (10.3 ± 3.4 vs. 4.4 ± 1.4, *p* < 0.05), IL12/IL10 (2.39 vs. 3.4 *p* = 0.1) and IL2 (1.3 ± 0.42 vs. 0.6 ± 0.3)
Derosa et al. [29]	NSCLC and RCC	64	Microbiome	Observational	Outcome (OS and PFS)	ATB vs. no ATB in RCC: increased risk of PD (75% versus 22%, *p* < 0.01), shorter PFS [median 1.9 vs. 7.4 mos, HR 3.1, 95% CI 1.4–6.9, *p* < 0.01], and shorter OS (median 17.3 vs. 30.6 mos, HR 3.5, 95% CI 1.1–10.8, *p* = 0.03). NSCLC: PD (52% versus 43%, *p* = 0.26) but decreased PFS (median 1.9 vs. 3.8 mos, HR 1.5, 95% CI 1.0–2.2, *p* = 0.03) and OS (median 7.9 vs. 24.6 mos, HR 4.4, 95% CI 2.6–7.7, *p* < 0.01).
Rolleret al. [30]	Colon cancer	37	Microbiome	Double blind	Immune cells	Increased mean IL-2 (221 ng/L vs. 132 ng/L) and IFNγ (1071 vs. 712 ng/L)
Botticelli et al. [31]	NSCLC	11	Microbiome	Observational	Immune cells	Tridecane and 2-pentanone associated to early progression (respectively *p* = 0.032 and *p* = 0.016). Fatty acids, lysine and nicotinic acids associated to long term beneficial effects of therapies (respectively *p* = 0.016, *p* = 0.032 and *p* = 0.016),
Routy et al. [32]	NSCLC and RCC	100	Microbiome	Observational	Immune cells	Increased PFS in presence of CD4+ and CD8+ against A. muciniphila and E. Hirae (*p* = 0.031 and *p* = 0.044 respectively)
Peters et al. [33]	Melanoma	27	Microbiome	Observational	Immune cells	Longer PFS (HR 95% CI) = 0.97 (0.95, 1.00), *p* = 0.02; number of shotgun subspecies: HR (95% CI) = 0.89 (0.79, 0.99), *p* = 0.03)
Gopalakrishnan et al. [34]	Melanoma	43	Microbiome	Observational Prospectic	Immune cells	PFS (HR = 2.95, 95% C.I. = 1.31–7.29, *p* = 0.03).
Matson et al. [35]	Melanoma	42	Microbiome	Observational Prospectic	Immune cells	Role of Microbial composition in R versus NR for this subset (*p* < 0.01)
Chaput et al. [36]	Melanoma	26	Microbiome	Observational Prospectic	Immune cells	Longer PFS (*p* = 0.0039) and overall survival (*p* = 0.051
Frankel et al. [37]	Melanoma	39	Microbiome	Observational Prospectic	Immune cells	Higher ICT responder if microbiomes is enriched with B. caccae (*p* = 0.032) and Streptococcus parasanguinis (*p* = 0.048)
Siska et al. [38]	RCC	54	Glycolysis	Observational	Immune cells	Higher PD-1highCD8+ T cells with hyperpolarized mitochondria and increased mitochondrial ROS and MTG staining (*p* < 0.05) and decreased PBMC PD-1lowCD8+ T cells cytoplasmic ROS (*p* < 0.05).
Ostadrahimi et al. [39]	Breast	30	Beta-glucano	Randomized, double blind, placebo controlled	Immune cells	Increased Global health status/QoL (*p* = 0.023)
Paixãoet al. [40]	Breast	45	n-3 fatty acids	Double blind randomized	Immune cells	Stable hsCRP in FG (initial median 0.1 (IQR 0.1–0.5), final median 0.3 (IQR 0.0–0.7), *p* = 0.510) vs. increased hsCRP in PG (initial median 0.1 (IQR 0.0–0.2), final median 0.2 (IQR 0.1–0.3), *p* = 0.024).

SD = stable disease; LT4 = CD4 Lymphocyte; LT8 = CD8 Lymphocyte; PGE2 = Prostaglandin E2; PFS: progression free survival; R = responders, NR = Non-responders; IQR = Interquartile range; hsCRP = high sensitivity C-reactive protein; FG = supplemented with fatty acids; PG = placebo group; RCC = renal cell carcinoma; mos = months, CI = confidence interval; HP = hazard ratio; NSCLC = non-small cell lung cancer; PD = primary progressive disease; ATB = antibiotics.

**Table 3:** Current ongoing trials.

Study Number	Target	Treatment	Evidence
NCT03072641	Colon Cancer	Probiotics	Randomized
NCT03048500	NSCLC	Metformin Hydrocloride + Nivolumab	Phase 2
NCT03311308	Melanoma	Metformin + Pembrolizumab vs. Pembrolizumab	Randomized double blind
NCT03048500	NSCLC	Metformin + Nivolumab	Randomized, Phase 2
NCT03314935	Advanced or Metastatic solid tumors	INCB001158 (Arginase inhibitors) + chemotherapy	Phase 1/2
NCT02903914	Advanced or Metastatic solid tumors	INCB001158 (Arginase inhibitors) +/− immune checkpoint therapy	Phase 1
NCT03047928	Melanoma	PDL1/IDO Vaccine + Nivolumab	Phase 1/2
NCT03291054	GIST	Epacadostat + Pembrolizumab	Phase 2
NCT01604889	Melanoma	Epacadostat + Ipilimumab	Phase 1/2 randomized, blinded
NCT02861300	Colon Cancer	CB-839 (oral glutaminase inhibitor) + Capecitabine	Phase 1/2
NCT03428217	Renal cell carcinoma	CB-839 (oral glutaminase inhibitor) + Cabozantinib vs. Cabozantinib	Phase 2, double blind randomized

NSCLC: non-small cell lung cancer. PDL1: Programmed death-ligand 1. IDO: Indoleamine 2, 3-Dioxygenase. GIST: Gastrointestinal stromal tumor.
